# Considerations on the application of enhanced recovery after surgery (ERAS) in the perioperative management of gastric cancer

**DOI:** 10.1097/JS9.0000000000003374

**Published:** 2025-09-08

**Authors:** Peiqin Zhan, Haihao Li, Jiansong Wang

**Affiliations:** Department of Urology, The Second Affiliated Hospital of Kunming Medical University, Kunming, Yunnan Province, China

*Dear Editor,*

We read with great interest the article published in the International Journal of Surgery by Ho-Jin Lee *et al*. The authors conducted a randomized, open-label, single-center controlled trial involving 92 patients scheduled for elective laparoscopic or robotic distal gastrectomy. These patients were randomly assigned to either an enhanced recovery after surgery (ERAS) program group or a conventional care group. The primary outcome was the quality of postoperative recovery, assessed using the Quality of Recovery-15 Korean version (QoR-15 K) on postoperative days 1, 2, and 3^[1]^. The study demonstrated that the ERAS group exhibited superior recovery quality, better pain control, reduced opioid use, faster gastrointestinal function recovery, and shorter hospital stays. This letter follows the TITAN Guidelines 2025 (regarding AI use transparency) and confirms zero utilization of AI tools in its preparation^[2]^.

According to the ERAS Society Guidelines, ERAS is an evidence-based, multidisciplinary perioperative management model designed to optimize preoperative preparation, intraoperative management, and postoperative rehabilitation. This approach aims to alleviate surgical stress, reduce complications, shorten hospital stays, and enhance patient satisfaction and functional recovery. The latest ERAS principles recommend the intake of clear liquids up to 2 hours before surgery and solid foods up to 6 hours preoperatively. However, this contrasts with older guidelines, such as the Chinese Medical Association’s Clinical Guidelines for Gastric Cancer (2021), which advocate for traditional preoperative fasting periods of at least 8–12 hours for solids and 6 hours for liquids. Such traditional practices not only increase patient discomfort and dehydration risks but also exacerbate stress responses, leading to more severe insulin resistance and disruption of perioperative homeostasis. Conversely, studies have confirmed that gastric cancer patients who consume 800 mL of a 12.5% carbohydrate solution 12 hours before surgery and an additional 400 mL 2–3 hours preoperatively experience reduced thirst, hunger, and anxiety, while also mitigating postoperative insulin resistance, hyperglycemia, and related complications. Regarding pain management, the NCCN Adult Cancer Pain Guidelines and the American Society of Anesthesiologists (ASA) 2018 Guidelines recommend the use of potent opioids for both non-general and general anesthesia analgesia, particularly in major or complex surgeries like gastrectomy. For patients with unstable conditions or cancer-related clinical progression, reducing opioid use may not be advisable. In contrast, the ERAS program and the Chinese Gastric Cancer Diagnosis and Treatment Guidelines (2022) emphasize minimizing opioid use in favor of multimodal analgesia strategies. Excessive opioid administration can lead to gastrointestinal side effects (e.g., nausea, vomiting, constipation), respiratory depression, immunosuppression, dependency, and delayed postoperative recovery or prolonged hospitalization. Furthermore, recent guidelines (e.g., the Chinese Gastric Cancer Guidelines and the Japanese Gastric Cancer Association) have increasingly discouraged routine placement of abdominal drains and nasogastric tubes, advocating instead for early oral feeding, which aligns with ERAS principles.

Upon careful review of Ho-Jin Lee *et al*’s article, we identified several limitations not addressed in the study. First, the lack of long-term follow-up data on postoperative quality of life, recurrence rates, and nutritional status limits the evaluation of ERAS’s impact on long-term clinical outcomes. Second, for cancer patients, recovery speed alone is insufficient; immune status, chemotherapy tolerance, weight changes, and psychological adaptation should also be considered. Third, the compliance rate with each ERAS component in clinical practice often falls short of expectations, potentially introducing variability that affects the consistency between research and clinical outcomes. Additionally, the following unaddressed covariates may influence the results: 1. Surgeon experience and technical variations (e.g., operative techniques, speed, attention to detail), which could affect postoperative recovery independently of ERAS. 2. Comorbidities such as diabetes, cardiovascular disease, and nutritional status (e.g., serum albumin levels), which may impact postoperative complication rates, wound healing, intestinal function recovery, and overall QoR scores. To enhance the robustness of the conclusions, we recommend incorporating multivariate regression or covariate adjustments in statistical models, collecting and controlling for nutritional scores (e.g., NRS-2002) and the Charlson Comorbidity Index, and conducting multicenter studies to account for surgeon and institutional variability.

We sincerely appreciate the authors’ outstanding contributions to exploring the efficacy of the ERAS program in postoperative recovery for gastric cancer patients and highly commend the significant value of their research.

## Data Availability

None.
